# Second Generation Amphiphilic Poly-Lysine Dendrons Inhibit Glioblastoma Cell Proliferation without Toxicity for Neurons or Astrocytes

**DOI:** 10.1371/journal.pone.0165704

**Published:** 2016-11-10

**Authors:** Jolanta Janiszewska, Inmaculada Posadas, Pablo Játiva, Marta Bugaj-Zarebska, Zofia Urbanczyk-Lipkowska, Valentín Ceña

**Affiliations:** 1 Institute of Industrial Research, Warsaw, Poland; 2 CIBERNED, Instituto de Salud Carlos III, Madrid, Spain; 3 Institute of Organic Chemistry, PAS, Warsaw, Poland; 4 Unidad Asociada Neurodeath, Facultad de Medicina, Universidad de Castilla-La Mancha, Albacete, Spain; Swedish Neuroscience Institute, UNITED STATES

## Abstract

Glioblastomas are the most common malignant primary brain tumours in adults and one of the most aggressive and difficult-to-treat cancers. No effective treatment exits actually for this tumour and new therapeutic approaches are needed for this disease. One possible innovative approach involves the nanoparticle-mediated specific delivery of drugs and/or genetic material to glioblastoma cells where they can provide therapeutic benefits. In the present work, we have synthesised and characterised several second generation amphiphilic polylysine dendrons to be used as siRNA carriers. We have found that, in addition to their siRNA binding properties, these new compounds inhibit the proliferation of two glioblastoma cell lines while being nontoxic for non-tumoural central nervous system cells like neurons and glia, cell types that share the anatomical space with glioblastoma cells during the course of the disease. The selective toxicity of these nanoparticles to glioblastoma cells, as compared to neurons and glial cells, involves mitochondrial depolarisation and reactive oxygen species production. This selective toxicity, together with the ability to complex and release siRNA, suggests that these new polylysine dendrons might offer a scaffold in the development of future nanoparticles designed to restrict the proliferation of glioblastoma cells.

## Introduction

Glioblastomas are the most common malignant primary brain tumours in adults and among the most aggressive and difficult-to-treat cancers [1,2]. Standard first-line treatment for glioblastoma patients includes surgery followed by focal fractionated radiotherapy with concomitant and adjuvant administration of the alkylating drug temozolomide [3]. However, glioblastomas invade the surrounding brain, making complete surgical excision highly improbable. As a result, glioblastoma patients have a poor prognosis, with a median survival of 14 months from diagnosis [4]. Thus, new therapeutic approaches are needed to treat this kind of tumour. One possible innovative approach involves the specific delivery of drugs and/or genetic material to glioblastoma cells where they can provide therapeutic benefits. Providing these benefits requires developing safe and efficient vectors that overcome the limitations posed by viral vectors (immunogenicity, insertional mutagenesis and high production costs) [5].

Dendrons are branched, monodisperse macromolecules with a well-defined molecular mass and structure, as well as a surface and/or intra-branch polyfunctionality that make these nanoparticles well suited for biomedical applications [6,7]. Amphiphilic dendrons of branched structure based on penta-functional core molecules obtained by functionalisation of positively charged basic amino acids like lysine, ornithine or β-amino-alanine, represent an interesting new group of molecules since they have a small size and are composed of natural molecules that might be more easily internalised than those based on synthetic molecules [8]. Among this group of dendrons, cationic polylysine (PLL) dendrons have already proven their usefulness in several biomedical applications, such as scaffolds for vaccine production or as carriers for the anticancer drug 5-fluorouracil [9].

Small interfering RNAs (siRNAs) are double-stranded RNA molecules that produce degradation in homologous mRNA [10]. The use of siRNA offers several advantages over other techniques used to diminish the levels of a specific protein, such as: ease of use, it is specific, it can inactivate any gene and it does not allow for compensatory pathways during development [11]. To achieve this, it is necessary to develop carriers that can insert siRNA into the target cells [12,13]. Different macromolecules or nanoparticles have been successfully used to deliver genetic material to different biological systems, including cell lines [14], primary cell cultures [15] or whole animals [16]. In addition, some of them have pharmacological actions by themselves [17] including antitumoural actions. Among the different nanoparticles, polymeric branched macromolecules, more specifically, dendrimers and dendrons, are among the most promising for future biomedical applications [18,19,20].

## Materials and Methods

### Synthesis

**Fig 1:**
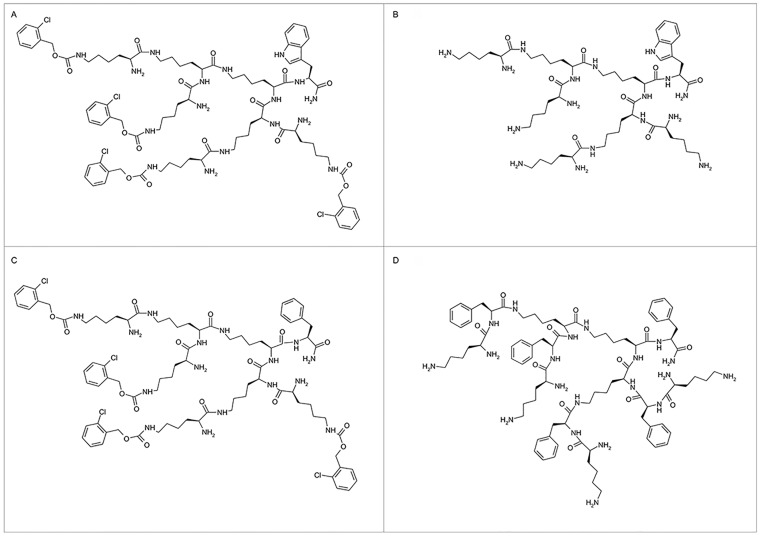
Chemical structure of dendrons. (A) F4; (B) F11; (C) F6, (D) F15.

To control the reaction efficiency, all the dendrons were successfully synthesised manually starting from 1 g of relatively common Rink Amide AM resin 200–400 mesh with a 0.71mmol/g loading capacity. A divergent approach was used to synthesise four amphiphilic dendritic amides based on trifunctional lysine monomer and using O-(7-azabenzotriazol-1-yl)-N,N,N',N'-tetramethyluroniumhexafluoro phosphate (HATU) as a coupling reagent. The raw products were purified by preparative high performance liquid chromatography (HPLC), after which their purity (monodispersity) was characterised by analytical HPLC and mass spectrometry (ESI-MS). Dendrimeric structure was confirmed on the basis of ^1^H and ^13^C NMR spectra that can be found in the Supporting Information section. The structures of the 4 dendrons are shown in Fig 1.

#### A. Preparation of Trp–• (R1) and Phe–• (R2), where –• denotes resin

Fmoc-protected Rink-resin (1 g; equiv. 0.71 mmol/g) was swollen in DMF for 15 minutes. The Fmoc group was removed using two five-minute treatments with 2:8 piperidine/DMF, and washed thoroughly with DMF. Once drained, the resin was acylated with a solution containing Fmoc-TrpOH (0.3633 g; 0.8520 mmol) or Fmoc-PheOH (0.3301 g; 0.8520 mmol), [2-(7-Aza-1H-benzotriazole-1-yl)-1,1,3,3-tetramethyluronium hexafluorophosphate] (HATU; 0.6479 g; 1.7040 mmol), N,N-Diisopropylethylamine (DIPEA; 0.4405 g; 3.4080 mmol) in anhydrous DMF for two hours at room temperature. After being drained and washed with DMF, the Fmoc group was removed with 20% piperidine in DMF for 15 minutes and washed with DMF.

#### B. Preparation of Lys–Trp–• (R3) and Lys–Phe–• (R4), where –• denotes resin

After draining resin **R1** or **R2**, the above acylation procedure was repeated for two hours with a solution containing Fmoc-Lys(Fmoc)OH (0.5032 g; 0.8520 mmol), HATU (0.6479 g; 1.7040 mmol), DIPEA (0.4405 g; 3.4080 mmol) in anhydrous DMF. After being drained and washed with DMF, the Fmoc group was removed with 20% piperidine in DMF for 15 minutes and washed with DMF.

#### C. Preparation of Lys–Lys(Lys)–Trp–• (R5) and Lys–Lys(Lys)–Phe–• (R6), where –• denotes resin

After draining resin **R3** or **R4**, the above acylation procedure was repeated for three hours with a solution containing Fmoc-Lys(Fmoc)OH (1.0064 g; 1.7040 mmol), HATU (1.2958 g; 3.4080 mmol), DIPEA (0.8810 g; 6.816 mmol) in anhydrous DMF. After being drained and washed with DMF, the Fmoc group was removed with 20% piperidine in DMF for 15 minutes and washed with DMF.

#### D. Preparation of Phe–Lys(Phe)–Lys[Phe–Lys(Phe)]–Phe–• (R7), where –• denotes resin

After draining resin **R6**, the above acylation procedure was repeated for three hours with a solution containing Fmoc-PheOH (1.3204 g; 3.4080 mmol), HATU (2.5916 g; 6.8160 mmol), DIPEA (1.7619 g; 13.6320 mmol) in anhydrous DMF. After being drained and washed with DMF, the Fmoc group was removed with 20% piperidine in DMF for 15 minutes and washed with DMF.

#### E. Preparation of dendrimer F4

After draining resin **R5**, the acylation procedure (described in point A) was repeated for three hours with a solution containing Boc-Lys(2-Cl-Z)OH (1.4140 g; 3.4080 mmol), HATU (2.5916 g; 6.8160 mmol), DIPEA (1.7619 g; 13.6320 mmol) in anhydrous DMF. The peptide-resin **(R8)** was deprotected and released by treatment with a TFA/H_2_O (98:2) solution at room temperature for one hour. The resin was filtered off, washed with ethyl acetate. The volatiles were then removed in vacuo, and the crude peptide **F4** precipitated twice with diethyl ether and collected by centrifugation. After decantation the product was solubilised in MeOH/H_2_O (1:1) and lyophilised. The crude peptide **F4** was purified by preparative HPLC using a C_18_ column, 250 x 21.20 mm, particle size 15 μm and a pore diameter of 300 Å. The mobile phase consisted of a gradient from 0 to 100% MeOH/H_2_O, 0.1% TFA, at a flow rate of 8.0 mL/min. The calculated mass was 1,773.75 g/mol (C_85_H_116_O_16_N_17_Cl_4_) and the yield 80%.

#### F. Preparation of dendrimer F6

After draining resin **R6**, the acylation procedure (described in point A) was repeated for three hours with a solution containing Boc-Lys(2-Cl-Z)OH (1.4140 g; 3.4080 mmol), HATU (2.5916 g; 6.8160 mmol), DIPEA (1.7619 g; 13.6320 mmol) in anhydrous DMF. The peptide-resin **(R9)** was deprotected and released by the procedure described in the point E. The crude peptide **F6** was purified by preparative HPLC (described in point E). The calculated mass was 1,735.72 g/mol; (C_83_H_116_O_16_N_16_Cl_4_) and the yield 80%.

#### G. Preparation of dendrimer F11

After draining resin **R5**, the acylation procedure (described in point A) was repeated for three hours with a solution containing Fmoc-Lys(Fmoc)OH (2.0130 g; 3.4080 mmol), HATU (2.5916 g; 6.8160 mmol), DIPEA (1.7619g; 13.6320 mmol) in anhydrous DMF. After draining and washing with DMF, the Fmoc group was removed with 20% piperidine in DMF for 15 minutes and washed with DMF. The peptide-resin **(R10)** was deprotected and released by the procedure described in the point E. The crude peptide **F11** was purified by preparative HPLC (described in point E). The calculated mass was 1,099.44 g/mol (C_53_H_96_O_8_N_17_) and the yield 64%.

#### H. Preparation of dendrimer F15

After draining resin **R7**, the acylation procedure (described in point A) was repeated for three hours with a solution containing Fmoc-Lys(Fmoc)OH (2.0130 g; 3.4080 mmol), HATU (2.5916 g; 6.8160 mmol), DIPEA (1.7619 g; 13.6320 mmol) in anhydrous DMF. After draining and washing with DMF, the Fmoc group was removed with 20% piperidine in DMF for 15 minutes and washed with DMF. The peptide-resin **(R11)** was deprotected and released by the procedure described in the point E. The crude peptide **F15** was purified by preparative HPLC (described in point E). The calculated mass was 1,650.10 g/mol (C_87_H_132_O_12_N_20_) and the yield 74%.

### Cell culture

Primary cortical neurons were obtained from E17 foetuses. Briefly, pregnant rats were obtained from a commercial source (Janvier Labs, Le Genest-Saint-Isle, France) and sacrificed the day of arrival by CO_2_ inhalation. The abdomen was open, the uterus exposed and the foetuses extracted. The culture was performed as previously described. Astrocytes were isolated from one-day old pups. Briefly, the pups were sacrificed by CO_2_ inhalation, the brain hemispheres were dissected and the meninges removed. The brain tissue was chopped with a scalpel blade and digested in trypsin (Gibco, Waltham, MA, USA) for 20 min at 37°C with gentle agitation. Digestion was stopped by adding 10% foetal bovine serum (Gibco, Waltham, MA, USA). Cells were collected by centrifugation, plated and incubated in Dulbecco’s modified Eagle medium (DMEM) supplemented with 2 mM L-glutamine, 20 units/mL penicillin, 5 μg/mL streptomycin and 10% heat-inactivated foetal calf serum. Cells were maintained at 37°C in a saturated humidity atmosphere containing 95% air and 5% CO_2_. The study was approved by the Committee on the Ethics of Animal Experiments from the University of Castilla-La Mancha (UCLM; protocol number PR-2014-10-12). Every effort was taken during the development of the work to minimize animal suffering. All animals were housed, treated and sacrificed in accordance with guidelines of the Ethics and Animal Experiments Committee from the University of Castilla-La Mancha (UCLM) and from European Union (directive 2010/63/EU) for the use of laboratory animals.

The C6 and U87 glioblastoma cell lines were obtained from ATCC (Manassas, VA, USA). The cells were carefully grown in DMEM supplemented with 2 mM L-glutamine, 20 units/mL penicillin, 5 μg/mL streptomycin and 10% heat-inactivated foetal calf serum. Cells were maintained at 37°C in a saturated humidity atmosphere containing 95% air and 5% CO_2_.

### Agarose gel retardation assay

Agarose gel electrophoresis was performed as previously described [21]. The dendron/siRNA complexes were prepared at increasing dendron/siRNA molar ratios and incubated for 30 min at room temperature. Electrophoresis was performed at 60 mV for 15 min, and the resulting gels were photographed under UV-illumination.

### siRNA release by poly-anion competition

siRNA release from dendrons after a challenge with the polyanion heparin was determined as a measure of the reversibility of siRNA binding in the presence of an excess of negatively charged compounds. Complexes were prepared as indicated above at a 3 μM dendron/100 nM siRNA molar ratio to ensure complete binding of siRNA by the dendron, and then incubated with increasing heparin sulphate concentrations ranging from 0.01 to 0.5 USP units. The samples were run on an agarose gel as described above.

### siRNA protection against RNAses

The protection provided by dendrons against RNAse-mediated cleavage was studied by incubating dendron/siRNA complexes prepared as indicated above at a 3 μM dendron/100 nM siRNA molar ratio or naked siRNA with 0.25% RNAse A (Sigma, Barcelona, Spain) for 30 minutes at 37°C. Afterwards, RNAse was inactivated by cooling the samples in an ice-cold water bath for 20 min followed by incubation at 4°C with heparin (0.5 USP units) to assure complete siRNA release from the nanoparticle as previously described [22]. Samples were then loaded onto an agarose gel, under the same experimental conditions as indicated above.

### Cellular siRNA uptake

Either C6 or U87 glioblastoma cells were cultured on glass coverslips as indicated above. A fluorescein-labeled siRNA (siRNA-FAM, Qiagen, Madrid, Spain) was incubated with the corresponding dendrons at a 3 μM dendron/100 nM siRNA molar ratio for 30 minutes under sterile conditions. The culture medium was changed to one containing the dendron/siRNA complexes and the cells incubated for different times ranging from 2 to 8 h. After washing, the cells were placed in a chamber located on the stage of an inverted fluorescence microscope (Nikon ECLIPSE, Tokio, Japan) and observed under a 40x oil immersion fluorescence objective. The samples were stimulated at a wavelength of 488 nm and the emitted fluorescence recorded at 520 nm using a commercial software (Metamorph, Molecular Devices, Sunnyvale, CA, USA). The percentage of glioblastoma cells taking up fluorescent dendriplexes (Dendron/siRNA-FAM) were determined by counting ramdomly selected microscopy fields and determining the intracellular fluorescence signal at 6 h. This time was established previously in preliminary experiments to be enough to determine siRNA-FAM uptake. No further increase in the percentage of cells taken up the siRNA-FAM was observed at 24 h. At least, 250 cells were analysed in every experiment that was repeated 3 times. Images were analyzed using ImageJ software [23].

### siRNA transfection and western-Blot analysis

Cells were incubated with dendrons alone or with dendriplexes formed by incubating dendrons (3 μM) with either a scramble siRNA or specific siRNA (100 nM; Sigma, Barcelona, Spain) for either rat or human p42-Microtubule-associated protein kinase (MAPK) for 30 minutes. Cells were treated for 72 hours, the medium was washed twice and the cells lysed. Western blots analysis were performed as previously described [24]. Briefly, protein content from cellular lysates was determined using the commercial kit “BCA protein assay” (Pierce, Rockfork, IL, USA). Protein samples (30–40 μg) were solubilized in sampling buffer containing (Tris-HCl (313mM, pH 6,8), SDS (10%), Bromophenol Blue (0,05%), Glycerol (50%) and 2-mercaptoethanol (5%), heated at 95°C for 5 min and loaded on 10% PAGE-SDS gels. Gels were transferred to nitrocellulose membranes (Bio-Rad Laboratories, Hercules, CA). Membranes were blocked in PBS-Tween 20 (0.1%) containing 5% non-fat dry milk and 0.1% BSA for 1 h at 4°C and subsequently incubated overnight at 4°C with a polyclonal anti-p42-MAPK antibody (1:1000) (Cell Signaling Techology, Beverly, MA) and, after removing the anti-p42-MAPK antibody, with a policlonal anti-β-actin antibody (1:4000) (Sigma Chemical Co., St. Louis, MO, USA) to correct for protein loading. Blots were then washed with PBS-Tween 20 (0.1%) and incubated with horseradish peroxidase (HRP)-anti-rabbit IgG (1:10.000) (Millipore, Bedford, MA, USA) for 2 h at 4°C. Immunocomplexes were visualized using an enhanced chemiluminiscence system (Millipore, Bedford, MA, USA). Densitometric analysis of immunoreactive bands was performed by using Quantity One Software (Bio-Rad Laboratories).

### Lactate dehydrogenase (LDH) assay

LDH toxicity assays were performed by measuring the release of lactate dehydrogenase (LDH) to the culture medium using the CytoTox96^®^ Non-Radioactive Cytotoxicity Assay kit (Promega, Madison, USA) following the manufacturer’s instructions as previously described [25]. LDH release was defined by the ratio LDH released/total LDH present in the cells, with the total LDH being 100%. All the samples were run in quadruplicate.

### Cell proliferation assay

The C6 and U87 glioblastoma cells were cultured in 24-well culture plates until 80% confluence was reached, and then either vehicle (bidistillated water, bdH_2_O) or dendron solution were added to the culture medium at the indicated concentrations for 72 h. The 5-Bromo-2’-deoxy-uridine (BrdU) Labelling and Detection Kit III (Roche, Basel, Switzerland) was used to analyse cell proliferation according to the manufacturer’s instructions as previously described.

### Mitochondrial transmembrane potential (Ψ_m_) measurement

The C6 and U87 glioblastoma cells were grown on poly-L-lysine-coated glass coverslips until 80% confluence was reached. The cells were then treated with vehicle (bdH_2_O) or the dendrons (3 μM) for different lengths of time. After the incubation period, the cells were washed twice in Krebs-Henseleit (K-H) solution with the following composition (in millimoles/litre): NaCl, 140; KCl, 5; CaCl_2_, 2; MgCl_2_, 1; Hepes, 10; glucose 11; pH 7.4). They were then incubated in K-H solution containing 4 nM tetramehtyl-rhodamine-methyl-ester (TMRM) (Molecular Probes, Carlsbad, CA, USA) for 20 minutes at 37°C in the dark. The cells were then washed twice with K-H solution and fluorescence was observed using a Nikon Diaphot (Nikon, Tokio, Japan) inverted microscope equipped with a 75-W Xenon lamp and a Nikon 40x, 1.3 numerical aperture, epifluorescence oil immersion objective. TMRM fluorescence was monitored using 535 nm excitation and 590 nm emission wavelengths every 10 s for 5 min. Afterwards, K-H solution containing 10 μM carbonylcyanide p-trifluoromethoxyphenylhydrazone (FCCP) was added to each preparation to dissipate the mitochondrial transmembrane potential (Ψ_m_). Images were acquired every 15 seconds for 5 minutes with a CCD camera (Orca 9100, Hamamatsu, Hamamatsu City, Japan) and analysed using commercial software (Universal Imaging; Molecular Devices; Toronto, Ontario, Canada). Linear regression fits of fluorescence data were obtained for each experimental condition and the slopes of the lines fitted by the least squares method were taken as the rate of loss of Ψ_m_ as previously described [26]. The n-fold changes in Ψm were calculated for the data obtained from the vehicle-treated groups.

### Reactive oxygen species (ROS) production

The C6 and U87 glioblastoma cells were grown on poly-L-lysine-coated glass coverslips until 80% confluence was reached. The cells were then treated with vehicle (bdH_2_O) or the dendrons (3 μM) for different lengths of time. After the incubation period, the cells were washed in Krebs-Henseleit (K-H) solution and incubated with 5-(and-6)-chloromethyl-2',7'-dichloro-dihydro fluorescein diacetate, acetyl ester (CM-H_2_DCFDA; 10 μM) (Molecular Probes, Carlsbad, CA, US) for 30 min at 37°C in the dark. The cells were then washed twice with K-H solution and superoxide production was monitored at room temperature on the stage of a Nikon Eclipse TE200 inverted microscope equipped with a 75-W xenon lamp and a Nikon 40X, 1.3 numerical aperture, epifluorescence oil immersion objective. Images were acquired with a CCD camera (Orca 9100, Hamamatsu, Hamamatsu City, Japan) and analysed using commercial software (Universal Imaging; Molecular Devices; Toronto, Ontario, Canada). The background was subtracted and fluorescence was recorded using a 535-nm excitation filter and a 635-nm emission filter. Frames were recorded every 15 s over a 10-min period. Fluorescence linear regression data were obtained for each condition and the slope of the best fitting line was taken as an index of superoxide production as previously described [27].

### Statistical analysis

The nonparametric variance analysis (Kruskal-Wallis), followed by Dunn's test, were used to evaluate the statistical differences between groups, with p<0.05 considered as statistically significant. These analyses were performed using SPSS 13.0 (SPSS, Chicago, IL).

## Results

### Dendron Synthesis

To control the reaction efficiency, all the dendrons were successfully synthesised manually starting from 1 g of relatively common Rink Amide AM resin 200–400 mesh with a 0.71 mmol/g loading capacity. A divergent approach to synthesise four amphiphilic dendritic amides based on a trifunctional lysine monomer was carried out using O-(7-azabenzotriazol-1-yl)-N,N,N',N'-tetramethyluroniumhexafluoro phosphate (HATU) as a coupling reagent. Raw products were purified by preparative high performance liquid chromatography (HPLC) and then their purity (monodispersity) was characterised by analytical HPLC and mass spectrometry (ESI-MS). In general, good yields of products with purities ranging from 64–80% and gram quantities of the products were obtained (0.914 to 1.346 g) showing that low generation peptide dendrons can be effectively synthesised by solid-phase methods using high-loaded resin.

### Dendron/siRNA interaction

**Fig 2:**
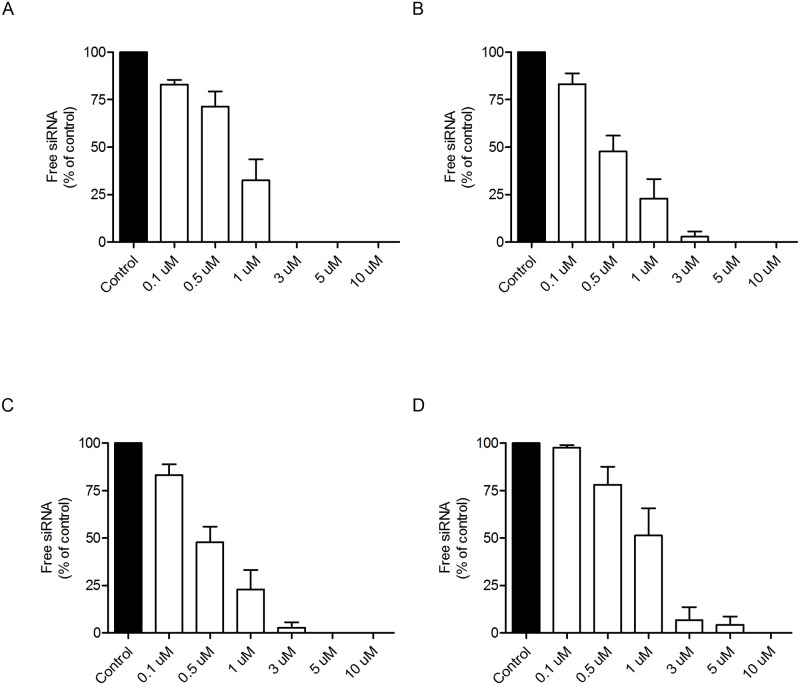
Gel retardation assay. siRNA (100 nM) was incubated with the different PLL dendrons at the indicated dendron/siRNA molar ratios at the top of each panel. The reaction mixture was loaded onto a 1.2% agarose gel as indicated in Material and Methods. Data show an experiment repeated twice with similar results. Panels represent the data for (A) F4, (B) F11; (C) F6 and (D) F15 dendrons.

**Fig 3:**
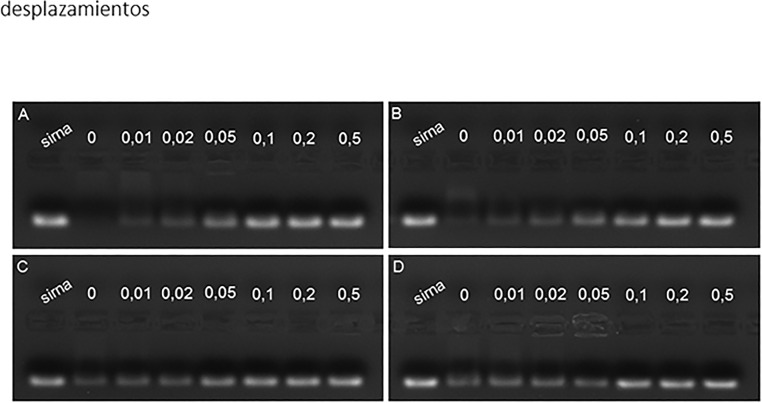
Heparin displaces siRNA from its binding to PLL dendrons. Dendriplexes were formed at a dendron/siRNA molar ratio of 30 being the siRNA concentration 100 nM. Following the dendriplex formation, siRNA was displaced from the dendrons by incubation with increasing heparin concentrations (0.01 to 0.5 USP units) and the samples run on a 1.2% agarose gel as indicated in Material and Methods. Panels show an experiment repeated twice with similar results. The panels represent the data for (A) F4, (B) F11; (C) F6 and (D) F15 dendrons.

All four PLL dendrons are positively charged at the pH (5.5) at which the experiments were performed. Accordingly, all of them were able to completely bind siRNA at varying dendron/siRNA molar ratios, being F4 the most efficient and F15 the less efficient (Fig 2). siRNA binding by the dendrons was reversible in the presence of increasing concentrations of the polyanion heparin, suggesting that the siRNA can be efficiently released from its dendron carrier. No significant differences were observed in the heparin’s efficiency at displacing the siRNA from the dendrons (Fig 3).

**Fig 4:**
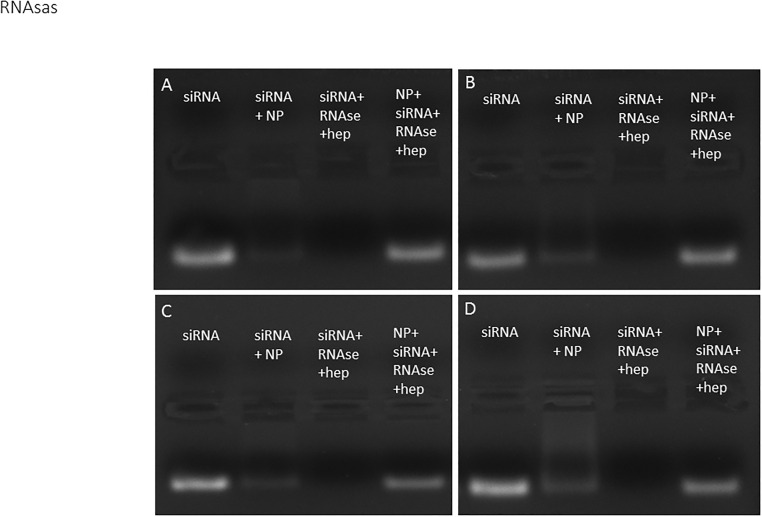
Polylysine dendrons protect siRNA from RNAse-mediated degradation. Dendriplexes (siRNA+NP) were formed at a dendron/siRNA molar ratio of 30 being the siRNA concentration 100 nM. The dendriplex or free siRNA were then exposed to 0.25% RNAse A for 30 min at 37°C. Following RNAase inactivation, bound siRNA was displaced from the dendrimer by 0.5 USP heparin units (hep) and the samples run on an 1.2% agarose gel. Panels show an experiment repeated twice with similar results. Panels represent the data for (A) F4, (B) F11; (C) F6 and (D) F15 dendrons.

**Fig 5:**
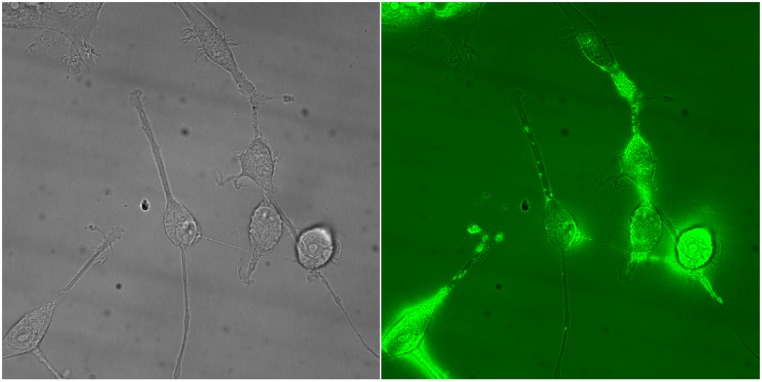
Uptake of FAM-siRNA by U87 glioblastoma cells. FAM-siRNA incorporation into U87 human glioblastoma cells after incubation for 8 hours with a dendriplex formed by 3 μM F6 dendron/100 nM FAM-siRNA. Images were acquired and analysed as indicated in Material and Methods. Left panel: bright field image; Right panel: Overlay of bright field and fluorescence images. Similar results were obtained for the 4 dendrons in both C6 and U87 glioblastoma cells after 4 and 8 hours of incubation.

**Fig 6:**
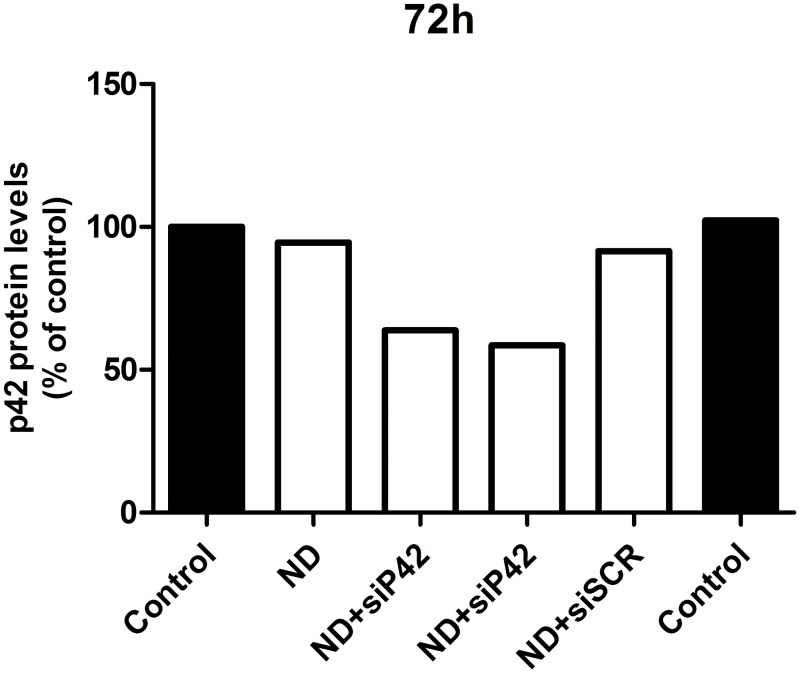
Dendron-mediated siRNA transfection. Glioblastoma C6 cells were treated for 72 hours with vehicle (Control); 3 μM F4 dendron (ND); dendriplexes formed by 3 μM F6 dendron/100 nM siRNA against p42-MAPK mRNA (ND+siP42) and dendriplexes formed by 3 μM F6 dendron/100 nM scramble siRNA (ND+siSCR). Cells were lysed and relative content of p42-MAPK relative to β-actin analysed as indicated in Material and Methods.

One of the requirements that needs to be fulfilled by a nanoparticle in order to be considered a good carrier for siRNA with possible biomedical applications is that it should be able to protect its siRNA cargo from the degradation caused by circulating RNases. All four PLL dendrons were able to protect the siRNA from RNAse-mediated degradation (Fig 4), and also to transport siRNA to the interior of both U87 and C6 glioblastoma cells (Fig 5). The number of cells taking up the dendron-bound fluorescent siRNA ranged from about 42% in the case of F6 to 51% in the case of F4 dendron. No differences were observed among dendrons or glioblastoma cell lines (S1 Table) When a specific siRNA directed against p42-MAPK mRNA was transfected to either C6 or U87 cells using the different dendrons, about 35 to 40% decrease in protein levels was observed for all the dendrons (Fig 6).

### Dendron-mediated cellular toxicity in glioblastoma cells

**Fig 7:**
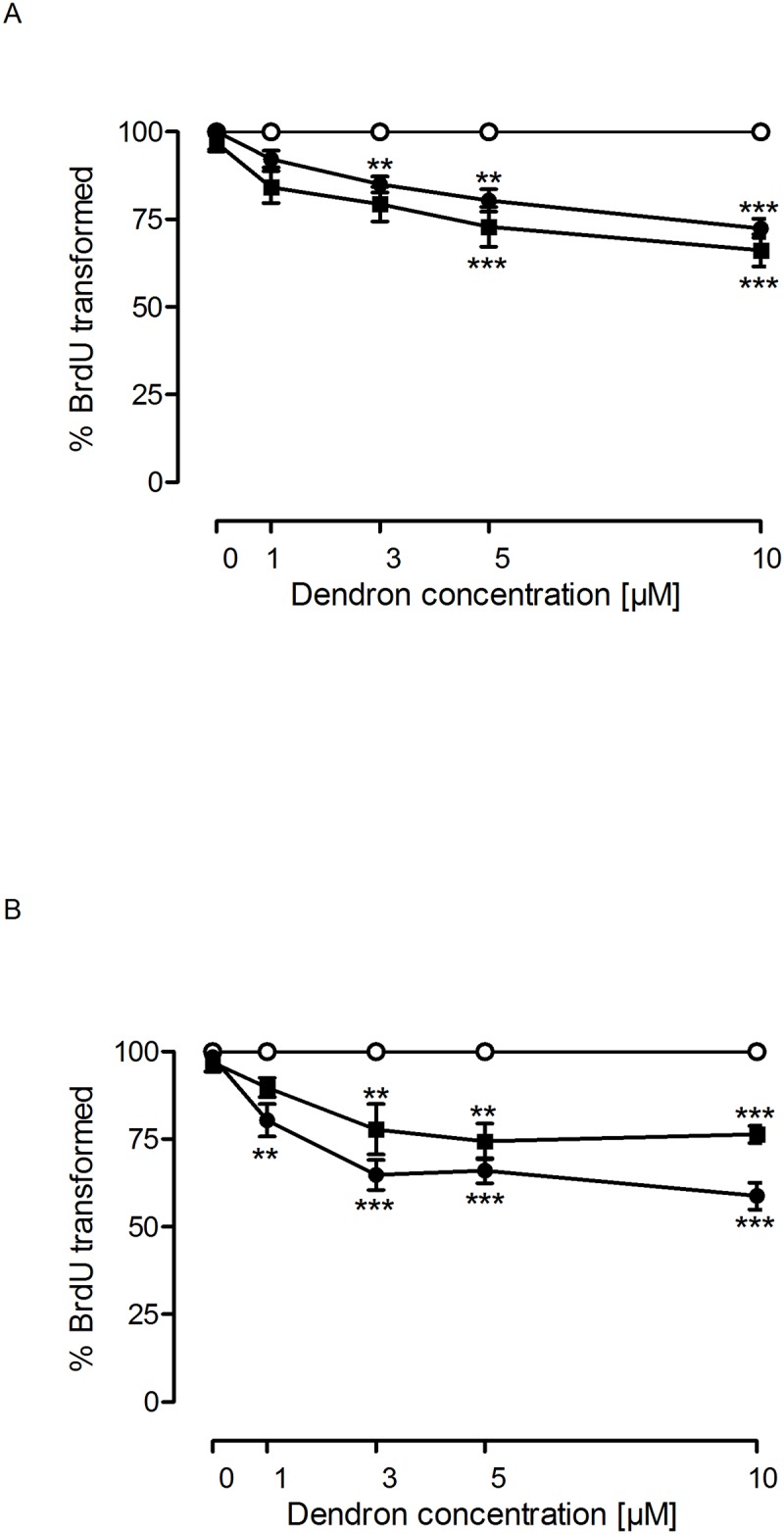
Dendron effect on glioblastoma cells proliferation. Either (A) C6 rat or (B) U87 human glioblastoma cells were exposed to vehicle (○; bdH_2_O) or increasing concentrations of dendrons F4 (●) and F11(■) for 72 h. Cell proliferation was measured as indicated in Material and Methods using BrdU incorporation. Data represent mean ± s.e.m. of 12 experiments. **p<0.05; ***p<0.01 when compared to vehicle.

**Fig 8:**
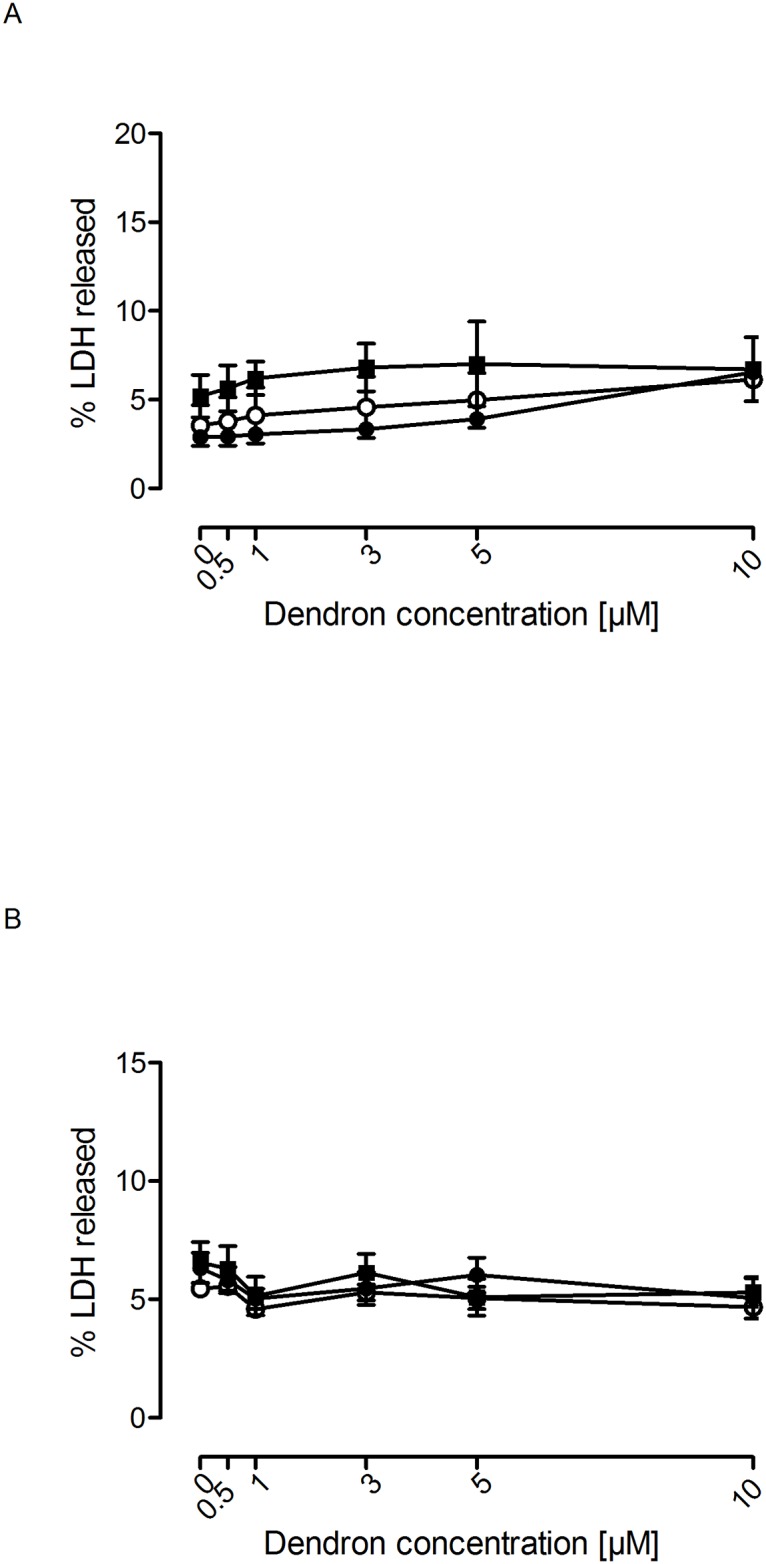
Lack of toxicity of dendrons F4 and F11 on neurons and astrocytes. Either (A) cortical rat neurons or (B) rat astrocytes were exposed to vehicle (○; bdH_2_O) or increasing concentrations of dendrons F4 (●) and F11(■) for 72 h. Cell death was measured as indicated in Material and Methods using LDH release as an index of cell death. Data represent mean ± s.e.m. of 10 to 12 experiments.

Increasing doses of the F4 and F11 dendrons caused a dose-dependent inhibition of cell proliferation in both murine (C6) and human (U87) glioblastoma cell lines (Fig 7). However, no significant toxic effect on glioblastoma cell proliferation was observed for F6 and F15 PLL dendrons (S1 Fig). The toxicity produced in these cells might represent a potentially useful therapeutic property against glioblastoma if the healthy cells of the CNS (i.e. neurons and astrocytes) were not affected. Therefore, we explored whether the dendrons were toxic to rat cortical neurons and astrocytes. As Fig 8 shows, no toxicity was observed in cortical neurons or astrocytes following exposure to F4 or F11 dendrons, indicating that this toxic effect affected only glioblastoma cells.

### Dendron-mediated free radical production in glioblastoma cells

**Fig 9:**
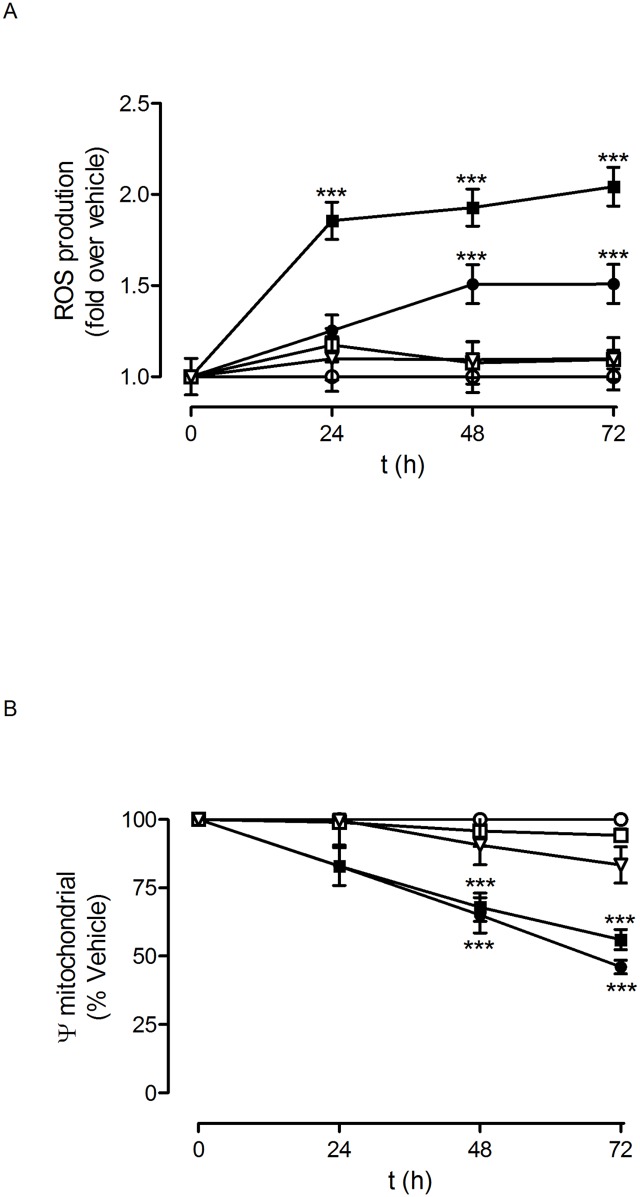
Dendron effect on C6 glioblastoma cell mitochondrial transmembrane potential and ROS production. C6 rat glioblastoma cells were exposed to vehicle (○; bdH_2_O) or increasing concentrations of dendrons F4 (●); F6 (□); F11(■) and F15 (▽) for the indicated times. Either (A) ROS production or (B) mitochondrial transmembrane potential were measured as indicated in Methods. Data represent mean ± s.e.m. of 20 to 24 cells. ***p<0.01. when compared to vehicle.

**Fig 10:**
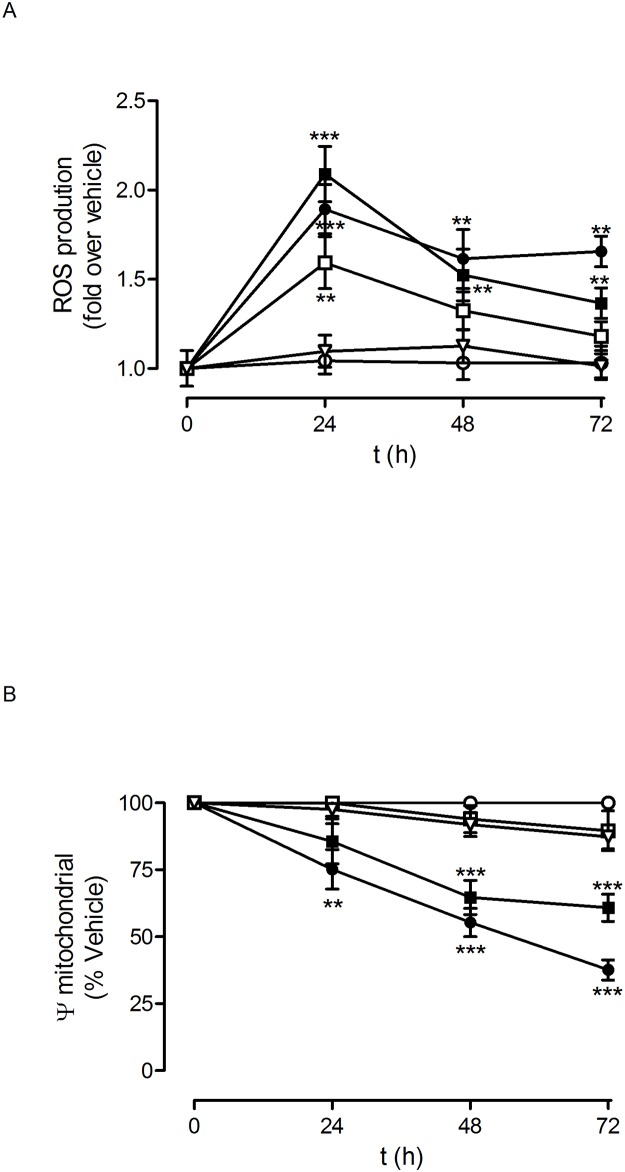
Dendron effect on U87 glioblastoma cell mitochondrial transmembrane potential and ROS production. U87 human glioblastoma cells were exposed to to vehicle (bdH_2_O; ○) or increasing concentrations of dendrons F4 (●); F6 (□); F11(■) and F15 (▽) for 72 h for the indicated times. Either (A) ROS production or (B) mitochondrial transmembrane potential were measured as indicated in Material and Methods. Data represent mean ± s.e.m. of 28 to 30 cells. **p<0.05; ***p<0.01 when compared to vehicle.

We decided to explore the molecular mechanisms involved in this toxic effect on glioblastoma cells. One of the possible toxicity mechanisms that can be considered therapeutic in the case of tumoural cells is the generation of free radicals (ROS), which can damage the cells by interacting with and inactivating key components of the cellular machinery [28]. This would lead to the production of different toxic effects, including inhibition of proliferation. It is well established that mitochondria play a relevant role in the genesis of ROS since mitochondrial depolarisation is generally linked to ROS generation [29]. Therefore, we explored whether there was a relationship between mitochondrial depolarisation and the observed toxicity produced by the PLL dendrons on glioblastoma cells. As shown in Figs 9B and 10B, PLL dendrons F4 and F11, which produced toxicity in both the C6 and U87 cell lines, also caused mitochondrial depolarisation. In addition to their effect on the mitochondrial potential of glioblastoma cells, dendrons F4 and F11 also caused an increase in ROS production, which peaked at 24 h and remained elevated for at least 72 h in the case of C6 (Fig 9A), while in U87 it also peaked at 24 h, but slightly decreased for the next 48 h (Fig 10A). This strongly suggests that the combination of mitochondrial depolarisation and an increase in intracellular ROS levels plays a key role in the observed inhibition of glioblastoma cell proliferation by dendrons F4 and F11.

It is interesting to note that dendron F15, at the highest concentration tested (10 μM), slightly decreased C6 cell proliferation while not affecting U87-cells proliferation (S1 Fig). This dendron did not modify ROS production or mitochondrial potential in either C6 (Fig 9) or U87 cells (Fig 10). On the other hand, dendron F6 did not affect either C6 or U87 glioblastoma cell proliferation (S1 Fig) or mitochondrial depolarisation (Figs 9B and 10B), although it caused a significant increase in ROS production only in U87 cells (Fig 10A).

## Discussion

Treating tumours that respond poorly to standard therapies requires new therapeutic approaches. One possibility involves delivering genetic material (likely siRNA) to block survival pathways, which would have a direct antitumoural effect, and/or enhancing the action of standard antitumoural drugs. Dendrimers and dendrons are one of the nanoparticles with the most potential for delivering siRNA to the CNS, which is highly relevant for the treatment of brain tumours like glioblastoma. The most significant advancement in dendron applications is their use as non-viral vehicles for introducing genetic material into cells. Recently, relatively small, scallop-seashell shaped dendrons were used as non-viral delivery systems for small interfering RNA (siRNA) either alone or docked in carbon-based nanomaterials [30,31]. Excellent transfection properties were also achieved when gold nanoparticles were covered with small, first-generation cationic dendrons [32].

The majority of the lower generation dendrons and dendrimers containing amino acids were obtained efficiently by solid-phase synthesis [33,34]. Despite the obvious advantages of this methodology, the reported milligram-scale quantity is often not sufficient for extensive biological testing. In comparison with growing linear peptide chains on polymer beads, dendritic peptides have a relatively compact structure and their surface groups, unprotected before the next coupling, are separated by a dendrimer tree. Therefore, the use of a high-loaded resin that yields dendrons in higher, preferably gram-scale, quantities is well justified. Problems associated with the solid-phase synthesis of a typical nonapeptide in various reaction conditions were recently reviewed [35]. Here we have used high-loaded Rink resin in the manual synthesis of a series of second-generation octapeptide dendrons and obtained about one gram for every dendron with a yield ranging from 64 to 80%.

Poly-lysine dendrons are biodegradable cationic polypeptides with good biocompatibility and low cytotoxicity that can self-assemble into protein-like globular structures in aqueous solutions [36]. The four PLL dendrons that we have synthesised possess an amphipathic structure and were constructed from small, branched monomers with either lipophilic (F4 and F6) or cationic (F11 and F15) surface, and hydrophobic tails (Phe or Trp). At the synthetic level, amphiphilicity in these molecules was achieved by orthogonal substitution of the available surface amino groups and modification of the C-terminus.

Positively charged dendrons [37,38] and their derivatives [39,40] have been previously used to deliver genetic material to different cell types. In addition, some dendrons and dendrimers, have shown intrinsic additional antitumoural properties, such as antiangiogenic actions [41], which makes them suitable molecules as scaffolds for antitumoural therapy. In addition, recent studies on peptide dendrimer/lipid mixtures used as effective transfection reagents also revealed the importance of appropriate charge distribution throughout the dendrimer tree, of the presence of active residues on the membrane/water interface, of the overall amphipathic character of the dendrimer and of the presence of co-lipids [42].

All four PLL dendrons synthesised here were able to efficiently bind siRNA and protect it from RNAse-mediated degradation. In addition, dendrimer-bound siRNA was displaced by the polyanion heparin, suggesting that siRNA dissociation from the dendron inside the cell could be easily achieved. Moreover, all four dendrons were able to facilitate the uptake of FAM-siRNA and to produce a decrease in protein levels of a protein when specific siRNA was transfected into glioblastoma cells, providing grounds for its possible use as a transfectant reagent [43]. However, the most interesting feature of two of these dendrons (F4 and F11) is their toxicity against glioblastoma cells (both human and murine), while sparing other CNS cells such as astrocytes and neurons that share the anatomical space with glioblastoma cells during the course of the disease. This is a very relevant property of these new dendrons since sparing healthy cells should be one goal when designing antitumoural drugs in general and, more specifically, for brain tumours where the ability to replace damaged healthy neurons is very limited. Small dendrimers terminated with similar polar aromatic residues were synthesized previously by our group and were characterized as potent but non-selective, membrane-active antimicrobials (Minimal Inhibitory Concentration in the micromolar range) [44]. As both, microbial and cancer cell membranes contain some excess of phospholipids with negatively charged heads testing was extended also towards glioblastoma cells. Moreover, several examples of natural antimicrobial peptides targeting both bacteria and malignant cells that emphasized the significance of indole moiety present in tryptophan (Trp or W) were reported [45]. In all cases amphiphilic, positively charged linear or cyclic peptides contained at least one Trp residue. Its position in the peptide sequence was critical for positioning and internalization of the peptide molecules inside the malignant cell cytoplasm and its affinity to mitochondrial membrane [46]. A prominent example is lactoferricin B, a natural cyclic peptide containing two Trp residues that was found to kill jurkat T-leukemia cells by mitochondria depolarization followed by cytochrome C release [47]. The inhibition of glioblastoma cell proliferation by F4 and F11 dendrons was directly related to mitochondrial depolarisation and to an increase in ROS production. Mitochondrial potential plays a key role in ATP synthesis, which is in turn required to maintain cellular homeostasis [48]. Its disruption by different noxious stimuli like ischemia or drugs leads to malfunctioning of the electron transport chain and might increase the production of free radicals, mainly superoxide anions. This leads to the production of peroxynitrites and hydrogen peroxide through a well-known mechanism [49]. These highly reactive compounds interact with different cell constituents which might be responsible for the observed inhibition of glioblastoma cell proliferation.

The absence of a significant effect from the two PLL dendrons (F6 and F15) on glioblastoma cell proliferation besides one of them (F6) having some effect on ROS production might be explained because, when ROS production increases, cancer cells might need, to function properly, the ATP synthesized by oxidative phosphorylation which is dependent on mitochondrial potential. In the absence of an increase in ROS production, cancer cells mostly use the glycolitic pathway instead of oxidative phosphorylation to metabolize glucose and to generate ATP [50], Therefore, it seems that the combined action of mitochondrial depolarization and ROS production (and not any one of them alone) is responsible for the observed dendron-mediated toxicity on glioblastoma cell proliferation which can be overcome by the glioblastoma cells when only either mitochondrial depolarisation or increased ROS production is present.

It is important to note that F4 or F11 PLL dendrons are only toxic to tumoural cells without affecting either neurons or astrocytes. The reason for this differential cellular toxicity is not clear, but it seems that the presence and position of Trp in the peptide sequence is critical for positioning the peptide molecules inside the malignant cell cytoplasm and its affinity to mitochondrial membrane.

In summary, in the present work, we have synthesized and characterised several new second generation amphiphilic PLL dendrons with varying distribution of hydrophobic and cationic centers and membrane-active indole residues located at the C-terminus. Two of them containing Trp residue at C-terminus show toxicity against two glioblastoma cell lines while being non-toxic to either neurons or astrocytes, two cell types that exist alongside tumoural cells in the CNS. We have found that the molecular basis for the differential toxicity of these nanomolecules to glioblastoma cells involve mitochondrial depolarisation and reactive oxygen species (ROS) production. In addition, the PLL dendrons might provide the possibility to vehiculise siRNA to the tumoural cells to selectively knock down key proteins involved in the survival and/or proliferation of the glioblastoma cells, providing an additional potential therapeutic approach to this tumour. These combined properties make these PLL dendrons good candidates to become scaffolds to develop future nanoparticles designed to restrict the proliferation of glioblastoma cells with no toxicity for healthy CNS cells.

## Supporting Information

S1 Fig Dendron effect on glioblastoma cells proliferation. Either (A) C6 rat or (B) U87 human glioblastoma cells were exposed to to vehicle (bdH_2_O; ○) or increasing concentrations of dendrons F6 (□) or F15 (▽) for 72 h. Cell proliferation was measured as indicated in Material and Methods using BrdU incorporation. Data represent mean± s.e.m. of 10 to 14 experiments. *p<0.05 when compared to vehicle.
(TIF)

S1 Table Percentage of cells taking up dendriplexes containing dendron and FAM. Cells (either C6 or U87 glioblastoma cells) were incubated for 6 hours with dendriplexes containing dendrons (3 μM) + siRNA-FAM (100 nM). The number of fluorescent cells was determined as indicated in Material and Methods. The data represent mean ± s.e. of the mean of 3 independent experiments. The number of cells counted in each individual experiment ranged from 253 to 325.
(DOCX)
