# Supplementary figures and images for: Second Generation Amphiphilic Poly-Lysine Dendrons Inhibit Glioblastoma Cell Proliferation without Toxicity for Neurons or Astrocytes
Source: PLoS One. 2016 Nov 10;11(11):e0165704. doi: 10.1371/journal.pone.0165704 (PMC5104433; doi:10.1371/journal.pone.0165704)

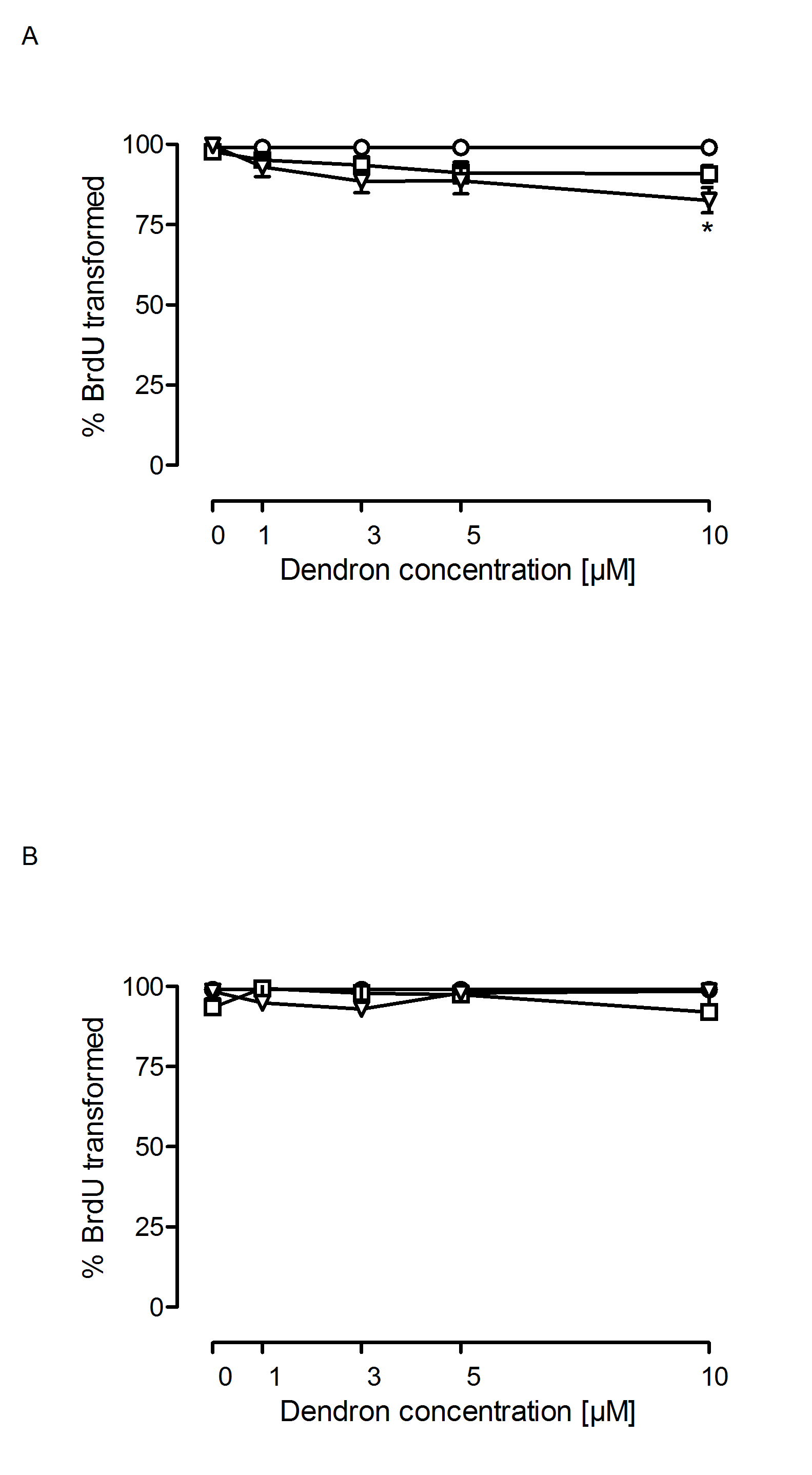

Supplement: S1 Fig — Either (A) C6 rat or (B) U87 human glioblastoma cells were exposed to to vehicle (bdH2O; ○) or increasing concentrations of dendrons F6 (□) or F15 (▽) for 72 h. Cell proliferation was measured as indicated in Material and Methods using BrdU incorporation. Data represent mean± s.e.m. of 10 to 14 experiments. *p<0.05 when compared to vehicle. (TIF) [file pone.0165704.s001.tif]
