# Supplementary material for: Second Generation Amphiphilic Poly-Lysine Dendrons Inhibit Glioblastoma Cell Proliferation without Toxicity for Neurons or Astrocytes
Source: PLoS One. 2016 Nov 10;11(11):e0165704. doi: 10.1371/journal.pone.0165704 (PMC5104433; doi:10.1371/journal.pone.0165704)
Supplement: S1 Table — Cells (either C6 or U87 glioblastoma cells) were incubated for 6 hours with dendriplexes containing dendrons (3 μM) + siRNA-FAM (100 nM). The number of fluorescent cells was determined as indicated in Material and Methods. The data represent mean ± s.e. of the mean of 3 independent experiments. The number of cells counted in each individual experiment ranged from 253 to 325. (DOCX) [file pone.0165704.s002.docx]

**Table S1. Percentage of cells taking up dendriplexes containing dendron and FAM.**

Cells (either C6 or U87 glioblastoma cells) were incubated for 6 hours with dendriplexes containing dendrons (3 µM) + siRNA-FAM (100 nM). The number of fluorescent cells was determined as indicated in Material and Methods. The data represent mean + s.e. of the mean of 3 independent experiments. The number of cells counted in each individual experiment ranged from 253 to 325.

| **Dendron forming the dendriplex** | **C6** | **U87** |
| --- | --- | --- |
| **F4** | 50.8+8.7 | 48.7+13.4 |
| **F6** | 42.1+9.9 | 48.8+11.3 |
| **F11** | 45.8+8.3 | 50.2+9.6 |
| **F15** | 48.7+7.1 | 50.6+12.3 |
